# Development of CD73 Inhibitors in Tumor Immunotherapy and Opportunities in Imaging and Combination Therapy

**DOI:** 10.1021/acs.jmedchem.4c02151

**Published:** 2025-03-19

**Authors:** Chunyang Bi, Jimmy S. Patel, Steven H. Liang

**Affiliations:** † Department of Radiology and Imaging Sciences, Emory University, Atlanta, Georgia 30322, United States; ‡ PharmaCenter Bonn, Pharmaceutical Institute, Pharmaceutical & Medicinal Chemistry, University of Bonn, An der Immenburg 4, D-53121 Bonn, Germany; § Department of Radiation Oncology, Winship Cancer Institute of Emory University, Atlanta, Georgia 30322, United States; ∥ Department of Biomedical Engineering, Emory University and Georgia Institute of Technology, Atlanta, Georgia 30322, United States

## Abstract

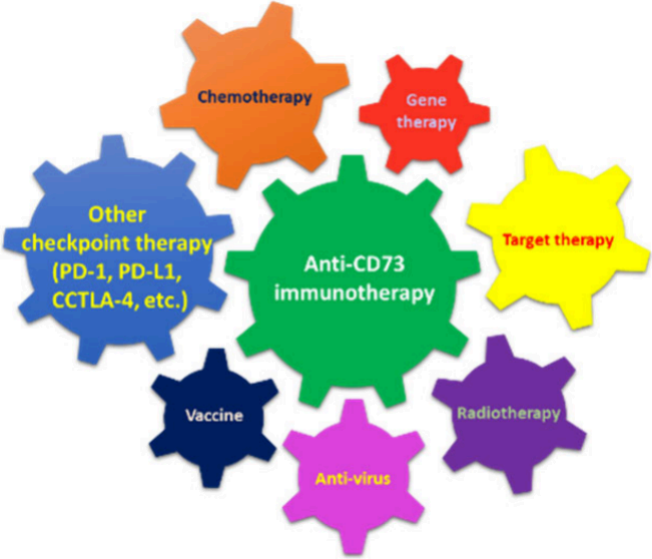

CD73 is a member of the membrane-bound enucleotidase family, which catalyzes the extracellular hydrolysis of adenosine monophosphate (AMP) to produce anti-inflammatory and immunosuppressive adenosine. As a novel checkpoint protein, CD73 is overexpressed in the immune system of various tumors, where adenosine is abundantly enriched. A large number of monoclonal antibodies (mAbs), nucleotides, and non-nucleotides as potent CD73 inhibitors are being discovered, providing opportunities for novel tumor immunotherapy. Currently, 18 CD73 inhibitors are in clinical trials, showing promising results in combination therapy for various solid tumors. The development of CD73-specific companion positron emission tomography imaging ligands holds potential for facilitating diagnosis, patient selection, and treatment efficacy evaluation throughout the entire process of CD73-targeted therapeutic development.

## Significance

Immunotherapy is now a key component in oncologic management. Diversifying immunotherapy by targeting various pathways will facilitate future patient care. Understanding the mechanism of CD73 inhibition is a key component by which hypotheses regarding the synergy with other systemic therapies may be generated. In addition, a review of current CD73 inhibitors may provide scientists with valuable insights to venture into untapped chemical space and advance drug discovery.

## Introduction

Immune checkpoint proteins play a pivotal role in regulating immune cell activation, differentiation, and function in response to various intrinsic and extrinsic stimuli.^1,2^ These proteins undergo stringent regulation, and any disruption in their expression can lead to significant alterations in the immune response, particularly affecting T cells within the tumor microenvironment (TME).^3,4^ Scientists have harnessed the mechanisms of various components of the immune checkpoint cascade, including cytotoxic T lymphocyte antigen 4 (CTLA-4), programmed cell death-1 (PD-1), and its ligand programmed cell death-ligand 1 (PD-L1), to develop novel immune checkpoint inhibitors (ICIs), thereby revolutionizing cancer immunotherapy.^5^ Since 2010, the FDA has successfully approved one CTLA-4 inhibitor (ipilimumab), three PD-1 inhibitors (nivolumab, pembrolizumab, and cemiplimab), and three PD-L1 inhibitors (atezolizumab, durvalumab, and avelumab).^6^

In recent years, cluster of differentiation 73, ecto-5′-nucleotidase (CD73) has emerged as a potential target for novel immunotherapies.^7−9^ CD73 is a surface enzyme that hydrolyzes extracellular adenosine monophosphate (AMP) yielding anti-inflammatory and immunosuppressive adenosine (ADO).^10,11^ It is expressed in various human tissues, where it maintains normal physiological functions such as resistance to pain, protection against inflammatory damage in the central nervous system (CNS), and ischemia–reperfusion injury in the brain, heart, liver, and kidney.^12^ The analysis of RNA-sequencing data from The Cancer Genome Atlas has revealed higher expression of CD73 in many (14) tumor specimens compared to adjacent normal tissues.^13^ This finding underscores the potential of CD73 as a novel checkpoint in restoring the antitumor immune response and supporting cancer therapy.^14−16^ For example, CD73 is widely targeted across different cancer types, including bladder cancer, lung cancer, breast cancer, gall bladder cancer, gastrointestinal cancer, colorectal cancer, prostate cancer, head and neck squamous cell carcinoma, pancreatic ductal adenocarcinoma, melanoma, glioblastoma, and chronic lymphocytic leukemia.^17,18^

**Figure 1:**
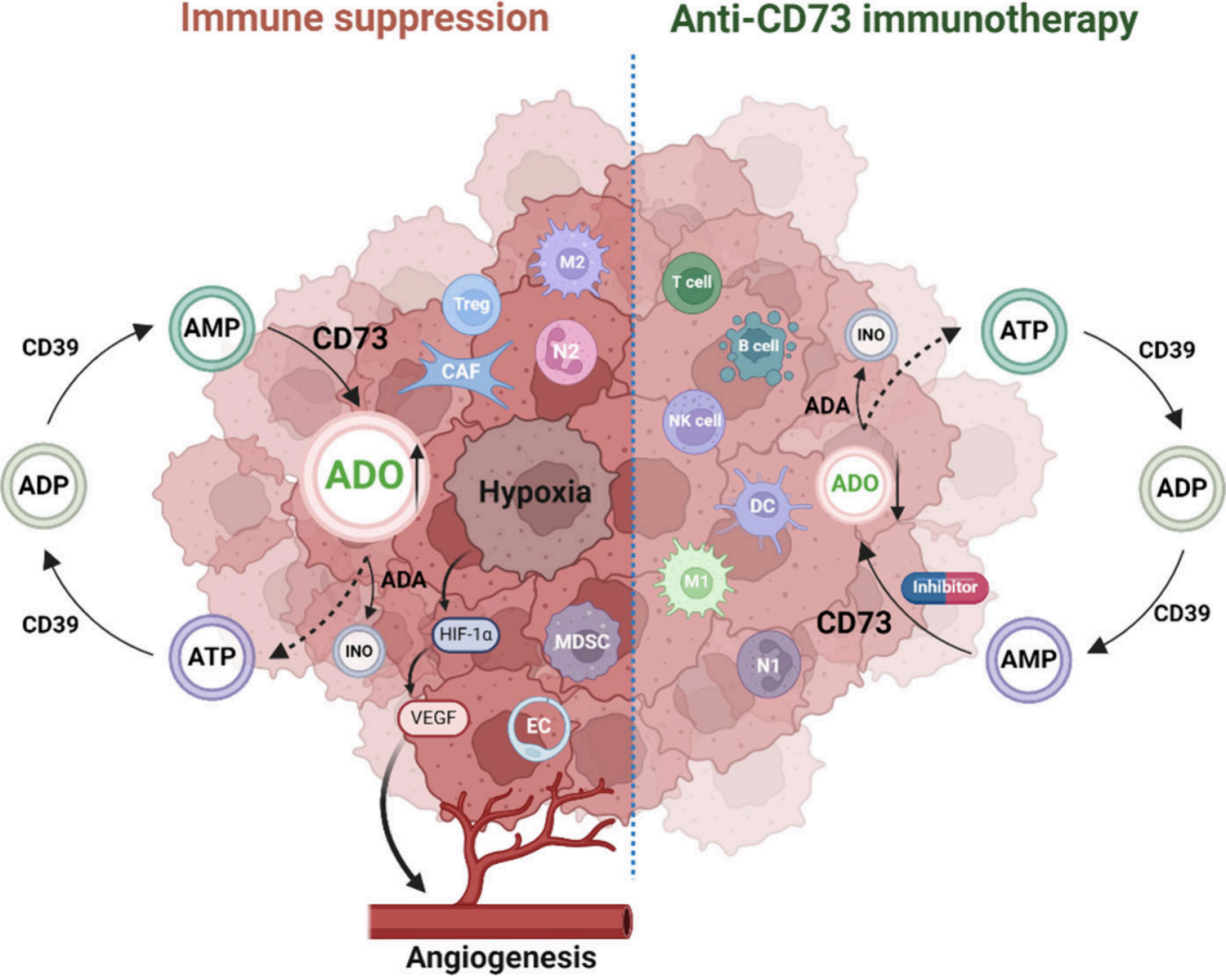
Immunotherapy targeting CD73 in the tumor microenvironment. Abbreviations: ADA, adenosine deaminase; ADO, adenosine; ADP, adenosine diphosphate; AMP, adenosine monophosphate; ATP, adenosine triphosphate; CAF, cancer-associated fibroblast; CD39, ectonucleoside triphosphate diphosphohydrolase-1; CD73, cluster of differentiation 73, ecto-5′-nucleotidase; DC, dendritic cell; EC, endothelial cell; HIF-1α, hypoxia-inducible factor-1α; INO, inosine; M1, M1 macrophage; M2, M2 macrophage; MDSC, myeloid-derived suppressor cell; N1, N1 neutrophil; N2, N2 neutrophil; NK cell, natural killer cell; Treg, regulatory T cell; VEGF, vascular endothelial growth factor.

Numerous studies have highlighted CD73’s significant negative role as a novel immune checkpoint protein within the tumor immune system (Figure 1).^19,20^ CD73 is highly expressed on the surface of endothelial cells in regulating vascular permeability and leukocyte trafficking in the bloodstream.^21^ While the absence of CD73 on endothelial cells in melanoma cells did not demonstrate a direct impact on tumor growth and metastasis, CD73 overexpression on these cells has been shown to impede T cell entry through the vascular wall and promote tumor angiogenesis.^22−24^ Hypoxia is a formidable inducer of ectonucleotidases in the TME via hypoxia-inducible factor-1α (HIF-1α), which is in charge of oxygen delivery and utilization, and the upregulation of HIF-1α directly promotes the expression of CD73 generating adenosine.^25^ Subsequently, the vascular endothelial growth factor (VEGF) is activated by HIF-1α to induce angiogenesis and suppress the immune response.^26^ Anti-CD73 therapy has been observed to effectively inhibit tumor angiogenesis and reduce the production of VEGF in a mouse model of breast cancer.^27^ Moreover, CD73 is also highly expressed on cancer-associated fibroblasts (CAFs), which constitute a major component of the reactive tumor stroma. CAFs secrete immunomodulatory factors, suppress T cell responses, and recruit M2 macrophages, myeloid-derived suppressor cells, and regulatory T cells (Tregs), thus playing a critical role in tumor progression.^28,29^ Inhibiting the overexpression of CD73 or other checkpoint proteins that interact with CAFs is increasingly recognized as a novel strategy for tumor immunotherapy. Over the past 3 years, more than 10 monoclonal antibodies (mAbs), bifunctional antibodies, and small molecules targeting CD73 have emerged. These agents are currently being investigated in clinical phase I, II, and III studies as novel immunotherapies for various oncologic diseases.

## 1 Reported Inhibitors

### 1.1 Nucleotides/Nucleosides

**Figure 2:**
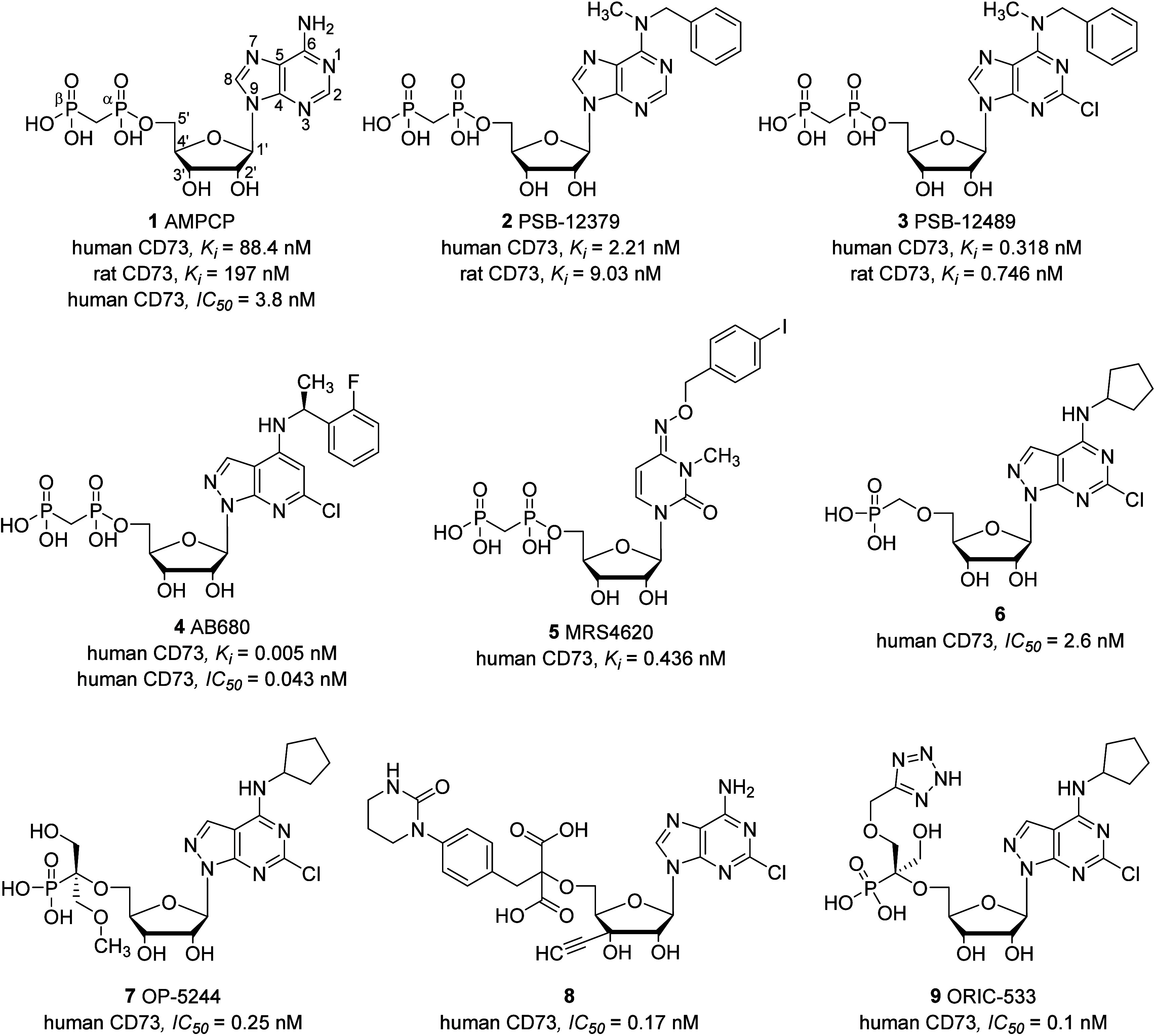
Representative nucleotide-derived CD73 inhibitors.

Since 2015,^30^ numerous highly potent CD73 inhibitors have been reported based on structure–activity relationships (SARs) around nucleotides. The majority of these nucleotide-derived CD73 inhibitors are competitive nucleoside methylenediphosphonates (AMPCP derivatives and analogs), including AMPCP (**1**),^30^ PSB-12379 (**2**),^30^ PSB-12489 (**3**),^31^ AB680 (**4**),^32^ and compound **5**.^33^ Additionally, compounds **6**,^34^ OP-5244 (**7**),^35^**8**,^36^ and ORIC-533 (**9**)^37^ have been identified as novel nucleotide-derived CD73 inhibitors (Figure 2).

Among the reported CD73 inhibitors, AB680 (**4**) based on the AMPCP scaffold^30,31,38^ has demonstrated potent activity with low clearance and a long half-life,^32^ making it a promising candidate, and it is currently undergoing evaluation in phase III clinical trials. Prior studies have also highlighted PSB-12379 (**2**) and PSB-12489 (**3**) as potent, selective, and metabolically stable CD73 inhibitors.^30,31^ Compound **5**, a pyrimidine nucleoside methylenediphosphonate derivative, was later identified as a novel CD73 inhibitor, exhibiting concentration-dependent inhibition *in vitro* against human head and neck squamous cell carcinoma.^33^ Replacement of the methylenediphosphonic acid moiety by both unmodified and modified methylenephosphonic acid moieties resulted in compounds **6** and OP-5244 (**7**), respectively. Incorporation of a relatively polar aryl moiety led to the development of compound **8**. These novel nucleotide-derived CD73 inhibitors have significantly expanded the repertoire of small-molecule CD73 inhibitors.^34,35^ For example, compound **6**, an analog of methylenephosphonic acid, demonstrates high potency, selectivity, low clearance, and a long half-life *in vivo*.^34^ Modification of compound **6** with hydroxymethylene and methoxymethylene groups at the α-position of the phosphonic acid yielded OP-5244 (**7**), which exhibited increased oral bioavailability and CD73 inhibition.^35^ OP-5244 demonstrated complete inhibition of adenosine production in both H1568 nonsmall cell lung cancer cells and CD8^+^ T cells in preclinical studies, and it modulated the AMP → ADO pathway to reverse immunosuppression *in vivo*.^35^ The most recent CD73 inhibitor, compound **8**, effectively suppresses AMP-mediated CD8^+^ T cells and tumor growth either as a monotherapy or in combination with chemotherapy (oxaliplatin, doxorubicin, or docetaxel) or a checkpoint inhibitor in preclinical *in vitro*/*in vivo* studies.^36^

Additionally, ORIC-533 (**9**) has emerged as a highly potent small-molecule inhibitor, surpassing the potency of AB680, and is currently in phase I trials as the first oral CD73 inhibitor for the treatment of relapsed or refractory multiple myeloma.^37^ As a new type of nucleotide, ORIC-533 has been reported to exhibit high metabolic stability with a half-life of 2.98 h in mice, and it features slow dissociation from CD73.^39,40^ These properties enable it to effectively restore cytokine secretion of CD8^+^ T cells and inhibit tumor growth when it is administered orally as a single agent.^41^

### 1.2 Overview of Structure–Activity Relationships of CD73 Nucleotide/Nucleoside Inhibitors

**Figure 3:**
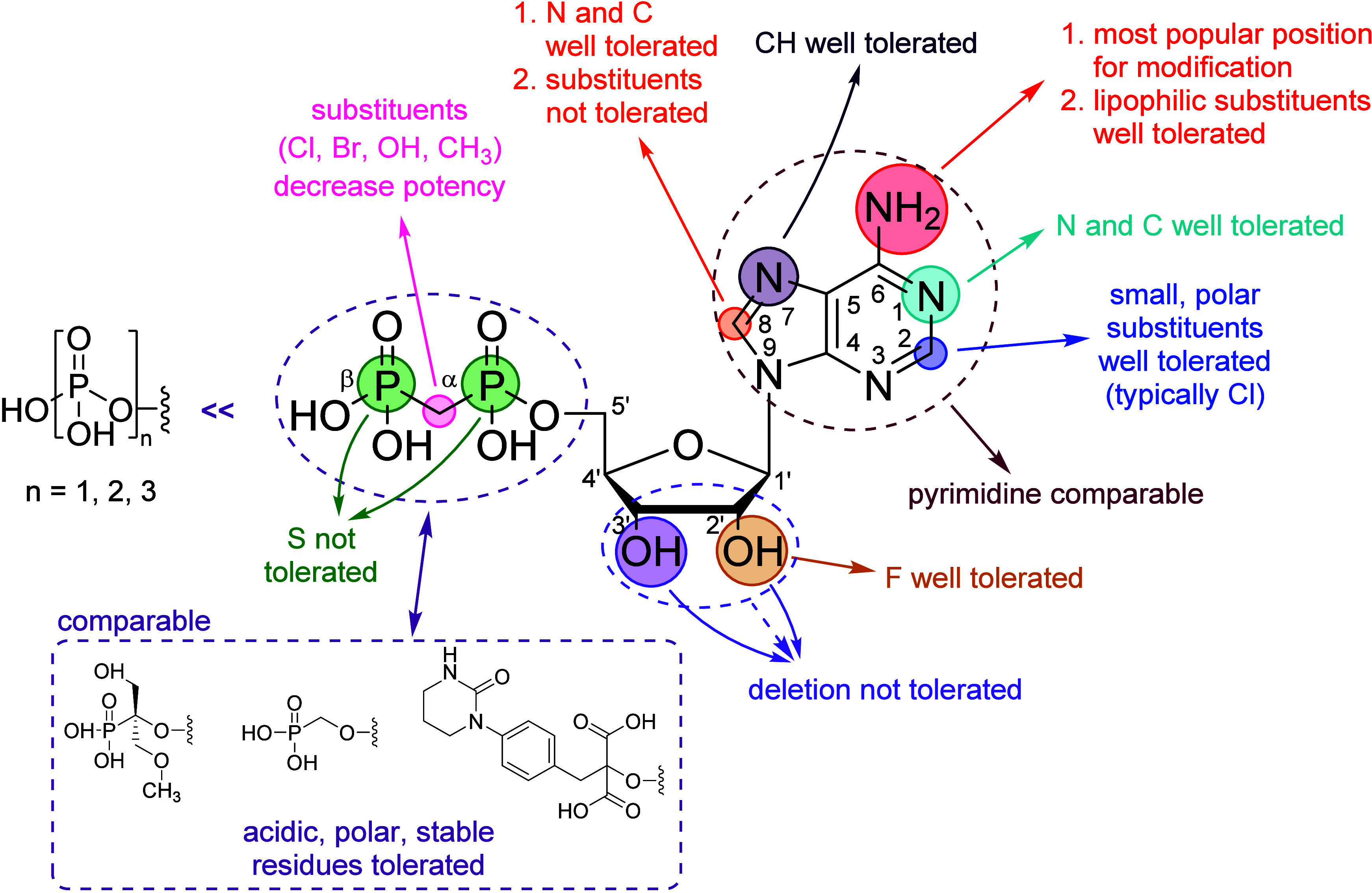
Summarized structure–activity relationships of the AMPCP-derived CD73 inhibitors.

AMPCP has served as a foundational scaffold for the development of CD73 inhibitors, as originally proposed by the Müller group, leading to numerous derivatives and analogs with *K*_i_ values in the low nanomolar range.^30−32,34,35^ A summary of the SAR analysis centered on AMPCP is depicted in Figure 3.

The 2-postion of the adenosine ring represents a crucial site for generating novel nucleotide inhibitors. In most instances, small and polar substituents such as halogens, CF_3_, CH_3_, OCH_3_, NH_2_, and NHNH_2_ are well-tolerated.^32,38^ While recent literature often favors Cl as the preferred substituent at the 2-position, further investigation is required to compare its efficacy against other halogens.^32,34−36^ Among all positions, the N^6^ position is frequently targeted for modification due to its synthetic accessibility and high inhibitory potency. Lipophilic substituents, such as 2-phenylethyl and cyclohexyl, are typically well-tolerated at the N^6^ position.^32,34^ Substitution of 1-N or 7-N with CH as well as the exchange of N and CH between 7- and 8-positions is generally tolerated, resulting in pyrazolopyrimidine nucleotides and pyrazolopyridine nucleotides that are comparable to adenine nucleotides.^32^ The highly potent pyrimidine nucleotide MRS4620 (**5**) represents a departure from the conventional AMPCP structure, underscoring the comparability of pyrimidine nucleotides to adenine nucleotides.^33,42^ Deletion of any OH residue or the entire glycol at the ribose is not tolerated, but substitution of the 2′-OH residue with a fluorine atom is well-tolerated.^42−44^

The phosphonate moiety plays a pivotal role in maintaining the potency of nucleotide-based CD73 inhibitors, with a majority of SARs involving at least one phosphate group. Notably, the activity of methylenediphosphonate is markedly superior to mono-, di and triphosphates.^30,45^ This discrepancy is presumed to arise from the presence of a methylene linker between the α- and β-P atoms, leading to a relatively stable phosphate resistant to hydrolysis. Additional data have demonstrated that introduction of halogens (such as Br or Cl) or OH and CH_3_ residues at the methylene linker leads to decreased activity.^30^ On the other hand, the replacement of the 5-O′ atoms of potent AMPCP derivatives by the more stable CH_2_ may enhance their activity. Replacement of the α-P atom or both the α-P and β-P atoms with S significantly reduces the activity.^44^ Subsequent studies have extensively replaced the methylenediphosphonate moiety with other phosphates and acidic polar residues such as methylenephosphonate^34,35,46^ and malonate.^36,47^ Interestingly, the acidic residues generated in these substitutions appear to exhibit stability comparable to that of methylenediphosphonate.

Overall, combining different potent substituents from two or more CD73 nucleotide inhibitors proves to be an effective strategy for enhancing activity. Typically, this approach yields a nucleotide with superior activity compared to that of any single-substituted predecessor. In summary, the incorporation of lipophilic substituents at the N^6^ position, along with small and polar substituents at the 2-position of adenine nucleotides or pyrazolopyrimidine/pyrazolopyridine nucleotides, leads to the development of the most potent CD73 inhibitors, exemplified by AB680 and PSB-12489.^30−32,34,35^

### 1.3 Non-nucleotides

**Figure 4:**
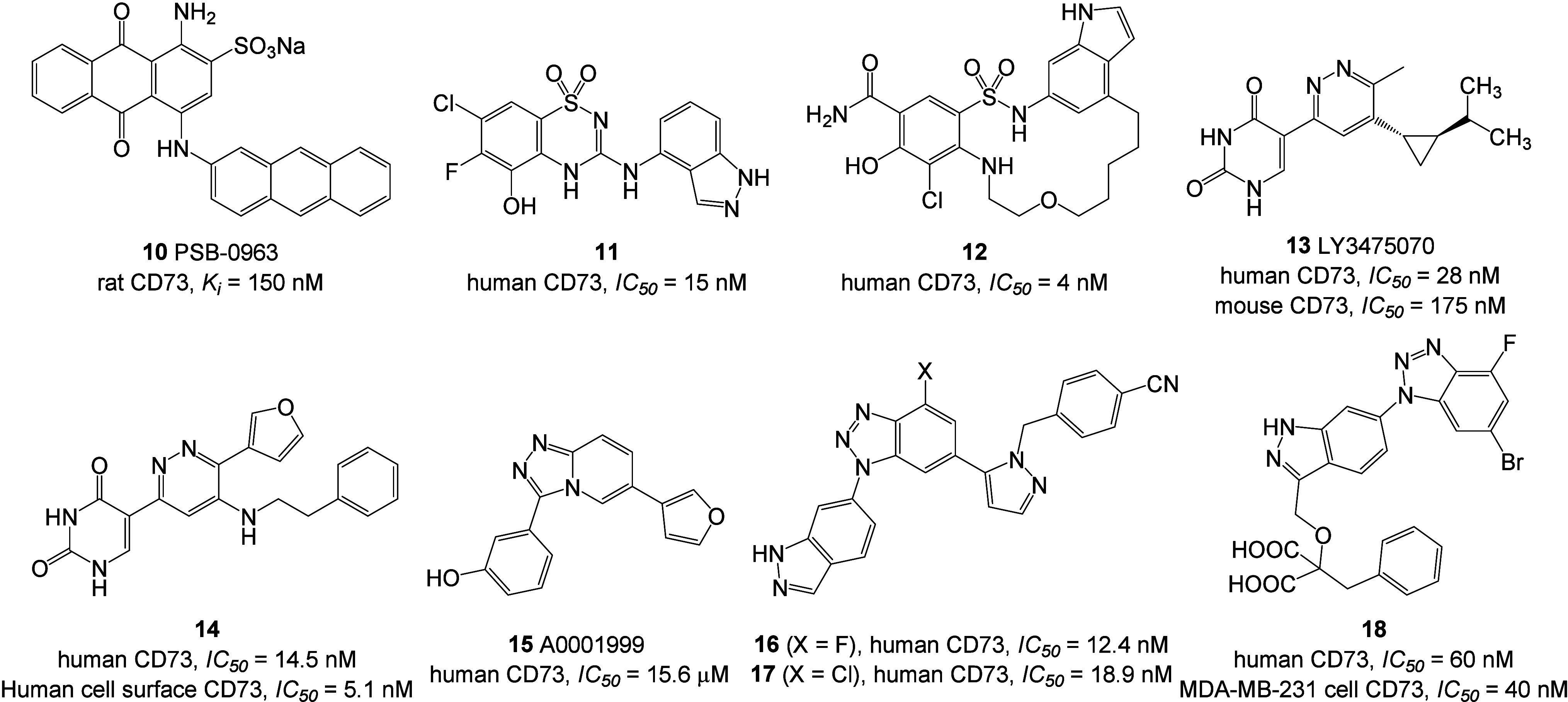
Representative non-nucleotide-derived CD73 inhibitors.

Currently reported non-nucleotide inhibitors have demonstrated a lesser degree of inhibition against CD73 compared to nucleotide-based inhibitors, with most exhibiting IC_50_ values in the micromolar range.^18,48^ However, the reduced acidity and improvements in membrane permeability of non-nucleotide CD73 inhibitors allow for formulations to enhance oral absorption. Furthermore, the moderate lipophilicity, electroneutrality, and relatively low molecular weight of most non-nucleotides enhance their potential for crossing the blood–brain barrier (BBB). Some representative non-nucleotide CD73 inhibitors with potent inhibitory activity include PSB-0963 (**10**),^49^ compounds **11**(50) and **12**,^51^ LY3475070 (**13**),^52^ and compounds **14**,^53^**15**,^54^**16**,^54^**17**,^54^ and **18**(55) (Figure 4).

The previous work of the Müller group demonstrated that the anthracenylamino-substituted derivative PSB-0963 (**10**) favorably inhibits CD73 and CD39 (*K*_i_ = 2.59 μM).^49^ In the SAR analysis, the sulfonate group was identified as an essential substituent of PSB-0963 for maintaining its anti-CD73 or anti-CD39 activity, prompting the subsequent synthesis of a series of PSB-0963 derivatives incorporating the sulfonate group.^56,57^ Compounds **11** and **12**, patented by GlaxoSmithKline (GSK), exhibit noteworthy activity, with compound **12** demonstrating superior performance among all reported non-nucleotide inhibitors.^50,51^ LY3475070, patented by Eli Lilly, represents the sole non-nucleotide CD73 inhibitor that has progressed to clinical study (phase I).^52^ In a recent study, LY3475070 was selected as a lead compound by the Lai group in the development of a series of CD73 inhibitors through various substituent modifications on the pyridazine ring of LY3475070.^53^ Among them, compound **14** emerged as one of the most potent uncompetitive inhibitors for tumor immunotherapy, demonstrating no obvious cytotoxicity, excellent metabolic stability (*t*_1/2_ = 3.37 h), and good oral bioavailability (*F* = 50.24%) *in vitro*/*in vivo*.^53^ The Lawson group identified the moderately potent CD73 inhibitor A0001999 (**15**) via high-throughput screening in a library of more than 200,000 compounds, and the following structure modifications generated two additional compounds, **16** and **17**, exhibiting high activity against CD73 but poor metabolic stability.^54^ Compound **17** was cocrystallized with human CD73, revealing a competitive binding mode.^54^ In further structure modifications by the Li group based on compounds **16** and **17**, compound **18** was generated, which exhibited improved metabolic stability (*t*_1/2_ = 1.2 h) and excellent efficacy and the reversal of immunosuppression when combined with the PD-L1 inhibitor KN035 in the mouse syngeneic lymphoma model.^55^

Compared to nucleotide modifications mainly based on the AMPCP scaffold, the development of non-nucleotide inhibitors allows greater flexibility in structural modifications. Although there is no comprehensive systematic summary of SARs for non-nucleotide-derived CD73 inhibitors, certain trends have nonetheless emerged. Hydrophilic pharmacophoric groups, such as carboxylic acids, sulfonates, and sulfonamides, have shown promising properties, particularly evident in CD73 molecular docking studies.^49,55,58^ For example, the acidic groups of compound **18** form ionic bonds with the dizinc catalytic center in the closed form of CD73, maintaining activity comparable to nucleotide inhibitors and implying that the adenosine backbone of nucleotide inhibitors can be replaced by bioactive acidic groups.^55^ The presence of key acidic groups has become a common characteristic between nucleotide- and non-nucleotide-derived CD73 inhibitors.

These extensive preclinical studies have enhanced the druggability of non-nucleoside inhibitors and have laid the foundation for the development of subsequent non-nucleoside-derived CD73 immunotherapy drugs. In addition, natural products and their analogs have also emerged as non-nucleoside-derived CD73 inhibitors, many of which contain an active carboxylic acid group with IC_50_ values in the micromolar range. Compounds such as betulinic acid, betulonic acid, and ZM557 may provide valuable structural insights for the design and development of new anti-CD73 inhibitors.^59,60^

Nevertheless, developing non-nucleotide CD73 inhibitors poses significant challenges related to structural targeting, selectivity, and pharmacokinetics. The active site of CD73 is highly optimized for binding its natural nucleotide substrate, AMP, making it difficult for non-nucleotide molecules to achieve effective inhibition without mimicking nucleotide structures. While targeting allosteric sites can circumvent this issue, identifying such sites requires a deep understanding of the enzyme’s conformational dynamics. Achieving selectivity is another critical hurdle, as inhibitors must avoid off-target effects on structurally similar enzymes, such as alkaline phosphatases, while maintaining efficacy across species for translational applications. Additionally, optimizing the pharmacokinetic properties of non-nucleotide inhibitors is particularly demanding, as these molecules must exhibit favorable ADME profiles while maintaining stability and, if necessary, the ability to penetrate the BBB.

## 2 Clinical Trials and Combination Therapy

To date, a total of 13 mAbs, including oleclumab, BMS-986179, CPI-006, NZV930, TJ004309, HLX23, AK119, IPH5301, PT199, Sym024, IBI325, CPI-006, and NZV930, along with one bifunctional antibody (dalutrafusp alfa) and four small molecules (quemliclustat, LY3475070, ORIC-533, and ATG 037), have advanced to clinical trials. These agents are being investigated either as monotherapies or in combination with other treatment modalities to target various cancers.

Compared to conventional chemotherapy and targeted therapies, immunotherapy has revolutionized cancer treatment and emerged as a cornerstone approach for various tumors.^9,61^ ICIs are widely utilized across different cancers due to their sustained reactivity and favorable efficacy. However, the response rate to ICI monotherapy is often limited, typically ranging from 20% to 40% overall response rate.^62^ At present, the majority of active CD73 trials involve combination with other cancer therapies, including immuno-oncology therapies, chemotherapies, targeted therapies, or radiotherapies. Among the registered clinical combination regimens involving CD73, approximately 89% incorporate the most utilized immuno-oncology therapies, with around 33% utilizing pembrolizumab, durvalumab, and nivolumab. Additionally, approximately 22% of these regimens involve chemotherapies such as paclitaxel, carboplatin, and gemcitabine.

The combination therapy of the anti-CD73 antibody oleclumab with the PD-L1 inhibitor durvalumab currently stands as the main antitumor combination therapy in a phase III study. This combination therapy was previously explored in a clinical phase II study as consolidation therapy for stage III nonsmall-cell lung cancer. Notably, the objective response rate (30.0%) and 12-month progression-free survival rates (62.6%) achieved with the combination therapy surpassed those of durvalumab monotherapy (17.9% and 33.9%, respectively).^63^ In addition to combinations with CD73 antibodies, the incorporation of small-molecule CD73 inhibitors can remarkably augment immune therapies targeting other immune checkpoints such as PD-1 and CTLA-4.^9^ Furthermore, certain checkpoint combination therapies have demonstrated effectiveness against CD73. For example, Mittal et al. reported that combination immunotherapy involving an A_2A_ receptor antagonist (SCH58261) and immune checkpoint blockade (anti-CTLA-4, anti-PD-1, or anti-Tim-3 monoclonal antibody) exhibited greater potency in inhibiting high expression of CD73 on tumor cells compared to any monotherapy.^64^ A comprehensive compilation of clinical CD73 inhibitors and combination therapies can be found in Table 1.

**Table 1:** Clinical Study Summary of CD73 Inhibitors^a^

type	agent	combination agent	condition or disease	phase
Monoclonal antibody	Oleclumab (MEDI9447)	Durvalumab	Multicancer	I/II/III
			Pancreatic cancer	
		Gemcitabine	Pancreatic ductal adenocarcinoma	
		Osimertinib	Nonsmall cell lung cancer	
		AZD4635	Muscle-invasive bladder cancer	
		Paclitaxel	Squamous cell carcinoma	
		Carboplatin	Triple-negative breast cancer	
		Tremelilumab	Prostate cancer	
		MEDI 0562	Relapsed ovarian cancer	
		Nab-paclitaxel	Luminal B breast cancer	
	BMS-986179	Nivolumab	Advanced solid tumors	I/II
	NZV930	PDR001	Advanced malignancies	I
		NIR178		
		KAZ954		
	TJ004309	Atezolizumab	Advanced or metastatic cancer	I/II
		Toripalimab	Ovarian cancer	
	JAB-BX102	Pembrolizumab	Advanced solid tumors	I/II
	INCA 0186	Retifanlimab	Advanced solid tumors	I
		INCB106385		
	HLX23 (Withdrawn)	–	Solid tumor	I
	AK119	AK112	Advanced solid tumors	I/II
		AK104		
		Pemetrexed	Nonsmall cell lung cancer	
		Carboplatin	Colorectal cancer	
		Oxaliplatin	COVID-19	
		Irinotecan		
	IPH5301	Trastuzumab	Advanced solid tumors	I
	PT199	Tislelizumab	Advanced solid tumors	I
	Sym024	Sym021	Solid tumor malignancies	I
	IBI325	Sintilimab	Advanced solid tumor	I
	CPI-006	Ciforadenant	Advanced cancer	I/III
		Pembrolizumab	COVID-19	
Bifunctional antibody	Dalutrafusp alfa	mFOLFOX6 Regimen	Advanced solid tumors	I/II
		Gemcitabine		
	(GS-1423)	Nab-paclitaxel	Pancreatic cancer	
	(AGEN1423)	Botensilimab	Pancreatic ductal adenocarcinoma	
Small molecule	Quemliclustat (AB680)	Zimberelimab	Oligometastatic prostate cancer	I/II/III
		Nab-paclitaxel		
		Gemcitabine	Gastrointestinal malignancies	
		Etrumadenant	Pancreatic cancer	
		Domvanalimab	Nonsmall cell lung cancer	
		Cisplatin	Pancreatic ductal adenocarcinoma	
		Docetaxe	Advanced biliary tract cancers	
	LY3475070	Pembrolizumab	Advanced cancer	I
	ORIC-533	–	Relapsed or refractory multiple myeloma	I
	ATG 037	Pembrolizumab	Advanced solid tumors	I

a See https://clinicaltrials.gov/.

## 3 Potentials of CD73 PET Imaging

Positron emission tomography (PET) falls under the umbrella of radiopharmaceutical imaging and enables the noninvasive visualization of pathophysiological processes.^65−69^ In oncology, PET imaging is often used for tumor localization and staging as well as assessment of treatment response.^70,71^

In current clinical oncology studies focusing on CD73, there is a notable scarcity of reported radiopharmaceuticals for both imaging and radioimmunotherapy, indicating an urgent need in this area. Cho et al. reported that the uptake of ^11^C-labeled AMP was significantly higher (10- to 100-fold) in various cell lines compared to FDG. Of note, radiotracer uptake was significantly and positively influenced by CD73 expression.^72,73^ CD73 inhibitors such as AMPCP block the uptake of ^11^C-labeled AMP in a dose-dependent manner without affecting the uptake of labeled adenosine.^72^ This observation motivates the evaluation of target engagement of CD73 inhibitors against tumors through analyzing their effects on [^11^C]AMP uptake. The direct radiolabeling of CD73 inhibitors may present as a potential strategy to identify CD73-expressing tumors and to assess response to CD73-specific therapy. Furthermore, the development of specific PET ligands targeting immune checkpoint proteins like CD73 and PD-1 can noninvasively monitor systemic and intratumoral immune alterations, holding significant and expanding value in clinical settings.^74^

Nevertheless, there are several challenges involved in developing a CD73 radiotracer. Achieving high specificity to CD73 is critical to avoid off-target binding to similar enzymes, which can compromise the image acquisition. Therefore, precursors for radiolabeling must show some inherent selectivity and specificity toward CD73. In addition, pharmacokinetic optimization is essential to balance rapid blood clearance for reduced background noise with sufficient retention at the target site. High plasma protein binding can abrogate the tracer’s availability for effective imaging. Also, if imaging CD73 in the brain is required, the radiotracer must penetrate the BBB, a difficult task given the strict requirements of lipophilicity and molecular size. All of these may be challenging when considering the nucleotide-based structures of the most clinically advanced CD73 inhibitors. Finally, radiolabeling presents its own set of challenges, as syntheses must retain a level of efficiency to allow for timely production and administration to patients. We postulate that a discussion of the key structural aspects of CD73 inhibitors as outlined above may assist medicinal chemists with designing strategies to tackle these challenges.

## 4 Summary and Future Directions

Immunotherapy represents a groundbreaking approach to tumor therapy, primarily centered on ICIs and T cell therapies.^75,76^ However, there is an urgent need for the development of next-generation checkpoints and corresponding ICIs to address the apparent lack of a broader efficacy and tumor drug resistance. In the TME, adenosine serves as a key mediator of a successful antitumor immune response, with prognostic potential via adenosine quantification.^77^ CD73 is gradually emerging as a potential target in various oncology studies and has been utilized or considered as an effective clinical tumor biomarker for evaluating survival, tumor metastasis, and prognostic implication in various cancer immunotherapies.^78−80^ By analyzing the adenosine purinergic pathway and the mechanism of anti-CD73 immune efficacy in the TME, we can gain deeper insights into the relationship between CD73 and tumors and devise new diagnostic and therapeutic approaches.

According to the analysis of CD73 structures and published SARs of nucleotides, we have the opportunity to design and develop highly potent small-molecule CD73 inhibitors. Currently, a significant number of clinical studies focusing on CD73 are centered around mAb immunotherapies targeting various cancers, with emerging studies involving one bifunctional antibody (dalutrafusp alfa). Most of these clinical trials explore combination therapies with other modalities, such as other checkpoint immunotherapies, chemotherapies, targeted therapies, or radiotherapies. The analysis of CD73 combination therapies suggests that selecting different combination therapies at different stages of tumors could optimize therapeutic outcomes. PET imaging stands as a potent method for assessing treatment efficacy, offering precise observation of drug–tumor interactions and expediting drug development. The potential use of [^11^C]AMP as a radiotracer presents a promising avenue for sensitive PET tumor imaging in CD73 studies. Furthermore, highly potent CD73 inhibitors hold potential for development as PET tracers, enabling pharmacokinetic and biodistribution studies to screen drug efficacy and assess immunotherapy responses. As another example, a novel study demonstrated the development and evaluation of two fluorine-18-labeled PET tracers, [^18^F]PSB-19427 and [^18^F]MRS-4648, for imaging CD73 expression in aggressive cancers, such as triple-negative breast cancer and pancreatic cancer. The study demonstrated that [^18^F]PSB-19427 performed favorably over [^18^F]FDG in imaging the aforementioned cancers due to higher specificity and longer retention, suggesting its potential as a superior PET imaging agent for solid tumors and possibly therapy monitoring.^81^ Future developments in CD73 PET imaging are eagerly anticipated.

In all, we have overviewed and analyzed the current landscape of CD73-related inhibitors and their therapeutic potential and outlined future directions in CD73 research. CD73 presents as a promising target in immuno-oncology for cancer therapy. The discovery of novel, highly potent CD73 inhibitors holds the potential to expand the repertoire of drug candidates for tumor immunology research.

## Abbreviations

ADA: adenosine deaminase
ADO: adenosine
ADP: adenosine diphosphate
AMP: adenosine monophosphate
AMPCP: adenosine-5′-*O*-[(phosphonomethyl)phosphonic acid]
ATP: adenosine triphosphate
BBB: blood–brain barrier
CAF: cancer-associated fibroblast
CD39: ectonucleoside triphosphate diphosphohydrolase-1
CD73: cluster of differentiation 73, ecto-5′-nucleotidase
CNS: central nervous system
CTLA-4: cytotoxic T lymphocyte antigen 4
DC: dendritic cell
EC: endothelial cell
FDA: U.S. Food and Drug Administration
GSK: GlaxoSmithKline
HIF-1α: hypoxia-inducible factor-1α
ICI: immune checkpoint inhibitor
INO: inosine
LAG-3: lymphocyte activation gene-3
M1: M1 macrophage
M2: M2 macrophage
mAb: monoclonal antibody
MDSC: myeloid-derived suppressor cell
N1: N1 neutrophil
N2: N2 neutrophil
NK cell: natural killer cell
PD-1: programmed cell death-1
PD-L1: programmed cell death-ligand 1
PET: positron emission tomography
RNA: ribonucleic acid
SAR: structure–activity relationship
TME: tumor microenvironment
Treg: regulatory T cell
VEGF: vascular endothelial growth factor
